# Impact of the SGLT2-inhibitor empagliflozin on inflammatory biomarkers after acute myocardial infarction – a post-hoc analysis of the EMMY trial

**DOI:** 10.1186/s12933-023-01904-6

**Published:** 2023-07-05

**Authors:** Martin Benedikt, Harald Mangge, Faisal Aziz, Pero Curcic, Sabine Pailer, Markus Herrmann, Ewald Kolesnik, Norbert J. Tripolt, Peter N. Pferschy, Markus Wallner, Andreas Zirlik, Harald Sourij, Dirk von Lewinski

**Affiliations:** 1 Department of Internal Medicine, Division of Cardiology, Medical University of Graz, Auenbruggerplatz 15, Graz, 8036 Austria; 2 Clinical Institute of Medical and Chemical Laboratory Diagnostics, Medical University of Graz, Graz, Austria; 3 Department of Internal Medicine, Division of Endocrinology and Diabetology, Medical University of Graz, Auenbruggerplatz 15, Graz, 8036 Austria; 4 Interdisciplinary Metabolic Medicine Trials Unit, Medical University of Graz, Graz, Austria

**Keywords:** Empagliflozin, Myocardial infarction, Inflammation, Interleukin-6, High-sensitive c-reactive protein

## Abstract

**Background:**

SGTL2-inhibitors are a cornerstone in the treatment of heart failure, but data on patients with acute myocardial infarction (AMI) is limited. The EMMY trial was the first to show a significant reduction in NTproBNP levels as well as improved cardiac structure and function in post-AMI patients treated with Empagliflozin compared to placebo. However, data on the potential impact of SGLT2-inhibitors on inflammatory biomarkers after AMI are scarce.

**Materials and methods:**

The EMMY trial is an investigator-initiated, multicentre, double-blind, placebo-controlled trial, which enrolled patients after AMI, receiving either 10 mg Empagliflozin once daily or placebo over a period of 26 weeks on top of standard guideline-recommended therapy starting within 72 h after percutaneous coronary intervention. In this post-hoc subgroup analysis of the EMMY trial, we investigated inflammatory biomarkers of 374 patients. The endpoints investigated were the mean change in inflammatory biomarkers such as high-sensitive c-reactive protein (hsCRP), interleukin-6 (IL-6), neutrophils, leukocytes, neutrophile/lymphocyte ratio (NLR) and platelet to lymphocyte ratio (PLR) from baseline to 26 weeks.

**Results:**

Baseline median (interquartile ranges) IL-6 was 17.9 pg/mL (9.0-38.7), hsCRP 18.9 mg/L (11.2–37.1), neutrophil count 7.9 x G/L (6.2–10.1), leukocyte count 10.8 x G/L (9.1–12.8) and neutrophile/lymphocyte ratio (NLR) of 0.74 (0.67–0.80). At week 26, a significant mean reduction in inflammatory biomarkers was observed, being 35.1 ± 3.2% (p < 0.001) for IL-6, 57.4 ± 0.7% (p < 0.001) for hsCRP, 26.1 ± 0.7% (p < 0.001) for neutrophils, 20.5 ± 0.6% (p < 0.001) for leukocytes, 10.22 ± 0.50% (p < 0.001) for NLR, and − 2.53 ± 0.92% for PLR (p = 0.006) with no significant difference between Empagliflozin and placebo treatment.

**Conclusion:**

Trajectories of inflammatory biomarkers showed a pronounced decline after AMI, but Empagliflozin treatment did not impact this decline indicating no central role in blunted systemic inflammation mediating beneficial effects.

## Introduction

Atherosclerosis (AS) is a chronic inflammatory disease of the arterial wall and, atherosclerotic plaques, when ruptured, are the primary underlying cause of acute myocardial infarction [1–3]. Cytokines like interleukin-6 (IL-6), adhesion molecules (VCAM-1, ICAM-1), and acute phase reactants (e.g. CRP, SAA) are centrally involved in the inflammatory atherosclerotic process [4, 5]. Growing evidence suggests that the immune system and especially T-cells are critical drivers and modifiers of AS pathogenesis [3]. Apart from this, high sensitivity (hs) CRP and IL-6 are useful markers for detecting inflammatory activity in AS. Hence, hsCRP is positively associated with blood cholesterol levels (LDL-cholesterol), risk of recurrent major adverse cardiovascular events (MACE), cardiovascular death, and all-cause mortality [6–8]. Higher plasma IL-6 and lower sIL-6R/IL-6 (soluble interleukin-6 receptor/interleukin-6) ratio early after ST-elevation myocardial infarction (STEMI) presentation are independently correlated with larger infarct size, reperfusion injury, and left ventricular dysfunction and a higher likelihood for left ventricular remodeling [9, 10]. IL-1 and IL-6 modifying agents are novel promising therapeutic strategies after myocardial infarction (CANTOS-Trial, VCUART3-Trial) [11–13]. These drugs have shown to decrease CRP levels and improve cardiovascular outcomes, but the underlying pathophysiologic mechanisms are not fully understood. Further, IL-6 is indirectly involved in cardiac remodeling via the TGFβ1/Smad signalling transduction pathway [13]. Apart from the molecular associations, specific blood cells, like neutrophils and leukocytes, were also identified to be an independent predictor for cardiovascular outcome showing positive correlations to infarct size and Troponin T levels, negative correlations were observed for the left ventricular ejection fraction (LVEF) [14–16]. Same finding were also revealed for the neutrophil to lymphocyte ratio as well as for the platelet to lymphocyte ratio demonstrating positive correlations to Troponin T levels [17] and were therefore suggested as predictors for adverse cardiac outcome [18–22]. Further, the PLR was identified to be correlated with the recurrence of myocardial infarction, stroke and subsequent heart failure and predicting long-term results in percutaneous coronary intervention in selecting patients with higher risk of no-reflow syndrome after PCI [23, 24].

On the other hand, the SGLT2-I AMI Protect registry showed that chronic application of sodium glucose linked transporter 2 inhibitors (SGLT2-I) significantly lowered inflammatory parameters at the time of admission and 24 h after AMI in patients with diabetes as compared to other oral anti-hyperglycaemic agents as a significant predictor of reduced inflammatory response (OR 0.457, 95% CI 0.275–0.758, p = 0.002) levels. Furthermore, patients on SGLT2-I therapy were found to have less hyperglycaemic events and smaller infarct size at admission compared to SGLT2-I naïve patients [25].

SGLT2-I therapy reduces the risk of cardiovascular death or hospitalization for heart failure in chronic heart failure with reduced ejection fraction (HFrEF), regardless of the presence or absence of diabetes [26–28]. Recently, two large outcome trials in patients with heart failure with mildly reduced (HFmrEF) and preserved ejection fraction (HFpEF) also reported a significant reduction in the combined endpoint of CV death and heart failure hospitalization [29, 30]. Based on these findings, SGLT2-I received a class I recommendation from the AHA/ACC/HFSA and ESC for the treatment of patients with HFrEF [31, 32] and recently maintained a class IIa recommendation in the American Heart Association (AHA) guidelines for the use in HFmrEF and HFpEF [32].

The EMMY trial showed a significant reduction in NTproBNP levels in patients with acute coronary syndrome for Empagliflozin compared to placebo, independent of diabetes status [33]. Nevertheless, data on the potential impact of SGLT2-I on inflammatory biomarkers in acute coronary syndrome are scarce.

This post-hoc analysis of the EMMY trial investigated inflammatory markers, their trajectories following acute myocardial infarction, their relation to functional cardiac parameters and the interplay with SGLT2-I.

## Materials and methods

### Study design

We conducted a post-hoc analysis of the recently published EMMY trial.

The EMMY trial was a 1:1 randomized, multicentric, investigator-initiated, double-blind, and placebo-controlled trial to investigate the potential effects of the SGLT2-I Empagliflozin 10 mg daily on structural (EDV, ESV) as well as functional (ejection fraction, E/E’) cardiac parameters and heart failure biomarkers (NTproBNP) in patients after AMI [33, 34]. We analysed specific inflammatory parameters like leukocytes and neutrophils, NLR, hsCRP and IL-6 at the clinical institute for medical and chemical laboratory diagnostics (CIMCL) of the Medical University of Graz.

The EMMY trial was approved by the relevant regulatory authorities, by the Ethics Committee of the Medical University of Graz, Austria (EK 29–179 ex 16/17; EudraCT 2016-004591-22) and registered on ClinicalTrials.gov (NCT03087773). The EMMY trial was fully conducted in conformity with the 1964 declaration of Helsinki and all subsequent revisions as well as in accordance with the guidelines laid down by the International Conference on Harmonization for Good Clinical Practice (ICH GCP E6 guidelines).

### Study cohort

From 2017 to 2022, we prospectively enrolled 476 patients with AMI undergoing percutaneous coronary intervention (PCI) in 11 study centres located in Austria Within 72 h after PCI, we randomly assigned hemodynamically stable patients either to receive Empagliflozin 10 mg daily or placebo added to the guidelines conformed therapy.

Exclusion criteria included other forms than type 2 diabetes mellitus (T2DM), acidosis (pH < 7.32), treatment with SGLT2-inhibitor within 4 weeks, recent urinary tract infection, as well as genital infections [33, 34].

### Clinical outcome

In this post-hoc analysis, the outcome variables were inflammatory biomarkers (leukocytes, interleukin-6, hsCRP, neutrophil granulocytes, neutrophil-to-lymphocyte ratio) defined as mean changes from baseline to 26 weeks. Blood samples were collected and centrally analysed from all study patients at baseline (randomisation), after 6 weeks (visit 2), and after 26 weeks (visit 4).

Explanatory variables in this post-hoc analysis include age, sex, treatment groups (Empagliflozin vs. placebo), hypertension, T2DM, smoking behaviour, NTproBNP, systolic function (LVEF), and diastolic function (E/E’), smoking status, body mass index (BMI), estimated glomerular filtration rate (eGFR), and lipid status. IL-6 and hsCRP were measured on the automated platform Cobas 8000, c-modul 702. The applied methods were the Elecsys IL-6 sandwichassay and Tina-quant C-Reactive Protein IV particle enhanced immunoturbidimetric assay technology (Roche Diagnostics, Mannheim, Germany).

### Statistical analysis

A complete case analysis of the inflammatory biomarkers in all patients participating in the EMMY trial with available frozen biomarker samples for all visits was performed. Baseline measurements were summarized using descriptive analysis with mean ± standard deviation (SD) or median with interquartile range (IQR) for continuous variables and frequency tables in percentage (%) for categorical variables.

A linear mixed effect model (LMEM) was established to analyse mean and percentage changes in inflammatory biomarker levels over visits including correlations with explanatory clinical variables (age, sex, T2DM, hyperlipidemia, smoking behavior, BMI, and hypertension) and biomarkers (functional cardiac parameters, eGFR, and NTproBNP). In simple LMEMs, we assessed correlations of inflammatory biomarkers with each explanatory variable over time. Significant associations observed in simple LMEM were included in the multiple LMEM along with treatment, visit, treatment-visit interaction, age, sex, and diabetes. The adjusted associations for multiple LMEM for each inflammatory marker were reported only for significant factors. The results were log-transformed for IL-6 and hsCRP in the graphical analysis. All statistical analyses were conducted in the Stata software version 17.0.

## Results

### Trial population

In the EMMY trial a total of 476 patients were successfully enrolled to either receive 10 mg Empagliflozin or placebo, a total of 374 patients (78,6%) with available frozen blood samples for all visits were included in the post-hoc analysis, 191 of them in the Empagliflozin group (51.1%) and 183 patients in the placebo group.

Baseline characteristics were comparable between both groups with an overall mean age of 57.6 ± 9.0 years, a mean body mass index (BMI) of 28.2 ± 4.3 kg/m² and 18.5% female patients. Analysis of the cardiovascular risk factors showed T2DM in 13.7% of all patients, a mean systolic blood pressure of 126.5 ± 13.34mmHg, and a mean diastolic blood pressure of 79.8 ± 8.3mmHg. A blood pressure control of below 140/90mmHg was achieved in 302 patients (80.75%) at baseline and 227 patients (60.70%) at week 26 **(**Table 1**)**. Arterial hypertension was present in 41.7% of patients, dyslipidaemia in 27.3%, and chronic nicotine abuse in 71.4%. LDL-cholesterol levels were 123.86 mg/dL ± 40.17 at baseline with 12 patients (3.31%) achieving an LDL-C target of < 55 mg/dL and 59.16 mg/dL ± 26.76 with 185 patients (50.68%) in the LDL target at week 26 **(**Table 1**)**. Positive past medical history for history of coronary artery disease (CAD) was reported in 8.0% of all patients, stroke in 1.3%, peripheral artery disease (PAD) in 1.3% and acute myocardial infarction in 4.3% (Table 2).

**Table 1:** Baseline Characteristics of EMMY trial participants stratified by treatment (N = 374)

Characteristics	EMMY cohort included in current analysis			
	All	Empagliflozin	Placebo	P-value
All, *n (%)*	374	191 (51.07)	183 (48.93)	--
Sex, *n (%)*				
Male	305 (82)	160 (84)	145 (79)	0.26
Female	69 (18)	31 (16)	38 (21)	
Age (years), median (IQR)	57 (52–64)	57 (52–64)	57 (52–65)	0.58
BMI (kg/m^2^), median (IQR)	27.7 (25.2–30.3)	27.7 (25.3–30.2)	27.7 (25.1–30.3)	0.86
Type 2 Diabetes, *n (%)*	51 (14)	24 (13)	27 (15)	0.54
Systolic BP (mmHg), median (IQR)	125 (117–131)	125 (115–131)	125 (118–131)	0.41
Diastolic BP (mmHg), median (IQR)	78 (74–85)	78 (74–85)	78 (74–85)	0.41
Smoking (active or former), *n* (%)	267 (71)	138 (72)	129 (70)	0.71
Dyslipidemia, *n (%)*	102 (27)	61 (32)	41 (22)	0.04
Hypertension, *n (%)*	156 (42)	73 (38)	83 (45)	0.16
CAD, *n (%)*	30 (8)	19 (10)	11 (6)	0.16
Stroke, *n (%)*	5 (1.3)	4 (2.1)	1 (0.6)	0.37
**Laboratory parameters**				
eGFR (mL/min/173m^2^), median (IQR)	92 (78–101)	93 (78–101)	90 (78–100)	0.68
Creatine kinase (U/L), median (IQR)	1648 (1201–2452)	1596 (1126–2478)	1669 (1257–2417)	0.43
Troponin T (ng/L), median (IQR)	3003 (2047–4647)	2947 (2062–4628)	3020 (1996–4871)	0.87
Total cholesterol (mg/dL), median (IQR)	192 (165–223)	192 (165–225)	191 (166–222)	0.98
LDL-cholesterol, (mg/dL), median (IQR)	122 (96–150)	122 (98–151)	123 (92–146)	0.82
HDL-cholesterol (mg/dL), median (IQR)	43 (36–52)	43 (36–52)	43 (36–52)	0.90
LVEF (%), median (IQR)	48 (43–54)	48 (43–53)	49 (43–55)	0.13
E/e‘, median (IQR)	9 (7–11)	9 (7–11)	9 (8–11)	0.56
NT-proBNP (pg/mL), median (IQR)	1365 (773–2192)	1271 (753–2127)	1436 (800–2217)	0.41
**Treatment**				
ACE-I/ARB, *n (%)*	361 (98)	186 (98)	175 (97)	0.67
Beta-blocker, *n (%)*	360 (96)	181 (95)	179 (98)	0.12
MRA, *n (%)*	143 (38)	70 (37)	73 (40)	0.52
Statin, *n (%)*	368 (98)	187 (98)	181 (99)	0.44
Ezetimibe, *n (%)*	43 (12)	23 (12)	20 (11)	0.74
Platelet inhibitory drugs, *n (%)*	374 (100)	191 (100)	183 (100)	> 0.99
Anticoagulation drugs, *n (%)*	26 (6.9)	11 (5.8)	15 (8.2)	0.35
Metformin, *n (%)*	37 (10.0)	17 (8.9)	20 (10.9)	0.51
GLP1-RA, *n (%)*	3 (0.8)	1 (0.5)	2 (1.1)	0.54

*BMI, body mass index; BP, blood pressure; CAD, coronary artery disease; AMI, acute myocardial infarction; PAD, peripheral artery disease; eGFR, estimated glomerular filtration rate; LVEF, left ventricular ejection fraction; NT-proBNP, N-terminal prohormone of brain natriuretic peptide; SD, standard deviation; IQR, interquartile range, LDL, low-density lipoprotein; HDL, high-density lipoprotein; ACE-I, angiotensin-converting enzyme inhibitor; ARB, angiotensin receptor blocker; MRA, mineralocorticoid receptor antagonist; GLP1-RA, glucagon-like peptide 1 receptor agonist*

**Table 2:** Overall control of blood pressure and LDL-C levels in all patients at baseline

Variables	Baseline	26 weeks	P-value
Systolic BP (mmHg), median (IQR)	125 (117–131)	130 (119–146)	< 0.001
Diastolic BP (mmHg), median (IQR)	78 (74–85)	81 (74–90)	0.002
Blood pressure < 140/90, n (%)	302 (81)	227 (61)	< 0.001
LDL-cholesterol, (mg/dL), median (IQR)	123 (96–150)	54 (43–69)	< 0.001
LDL-C < 55 mg/dl, n (%)	12 (3)	185 (51)	< 0.001

*BP, blood pressure; IQR, interquartile range, LDL-C, low-density lipoprotein-cholesterol*

At randomization, baseline median (interquartile range [IQR]) NTproBNP level was 1365 pg/mL (773–2192), median Troponin T was 3003 ng/L (2047–4647), median creatinine kinase was 1648 U/L (1201–2452) and median estimated glomerular filtration rate (eGFR) was 92.0 ml/min/1.73m² (78.1-100.7). Echocardiographic parameters showed a median (IQR) LVEF of 48.0% (43.0-53.7) and a median E/E’ of 9.1 (7.5–10.7) **(**Table 2**)**.

At baseline inflammatory biomarkers were increased in both groups (within 72 h after presentation with AMI) and equally distributed in both groups with a median IL-6 (IQR) of 17.9 pg/mL (9.0-38.7), a median hsCRP of 18.9 mg/L (11.2–37.1), a median neutrophil rate of 7.9 × 10^9/L (6.2–10.1), a median leukocyte rate of 10.8 × 10^9/L (9.1–12.8), a median NLR of 0.74 (0.67–0.80), and a median PLR of 125.58 (97.14-171.82) **(**Table 3**)**.

**Table 3:** Inflammatory markers at each visit and percentage change in markers over time

	Baseline median (IQR)	6 weeks median (IQR)	26 weeks median (IQR)	% change Mean ± SEM	% change (Empagliflozin - Placebo) Mean ± SEM	P-value
IL-6						
All	17.90 (9.00–38.70)	4.10 (3.20–5.70)	3.40 (2.70–4.60)	-35.13 ± 3.16		< 0.001
Empagliflozin	16.20 (8.70–34.70)	4.10 (3.10–5.40)	3.20 (2.70–4.30)	-33.72 ± 4.42	-2.88 ± 6.31	0.649
Placebo	19.50 (9.10–40.90)	4.10 (3.20–6.00)	3.40 (2.80–4.80)	-36.60 ± 4.52		
hsCRP						
All	18.85 (11.20–37.10)	1.15 (0.70–2.70)	0.80 (0.60–1.70)	-57.37 ± 0.71		< 0.001
Empagliflozin	17.80 (10.40–35.70)	1.20 (0.70–2.30)	0.80 (0.60–1.00)	-57.82 ± 1.00	0.92 ± 1.43	0.521
Placebo	21.40 (12.30–40.80)	1.10 (0.60–3.00)	0.90 (0.60–1.70)	-56.90 ± 1.02		
Neutrophils						
All	7.90 (6.20–10.10)	4.48 (3.50–5.50)	4.11 (3.31–5.01)	-26.09 ± 0.72		< 0.001
Empagliflozin	7.75 (6.27–9.75)	4.50 (3.60–5.40)	4.19 (3.35–5.00)	-25.82 ± 1.01	-0.56 ± 1.44	0.700
Placebo	7.90 (6.10–10.10)	4.40 (3.40–5.60)	4.00 (3.20–5.10)	-26.37 ± 1.03		
Leukocytes						
All	10.77 (9.10–12.80)	7.30 (6.26–8.65)	7.03 (5.87–8.37)	-20.46 ± 0.60		< 0.001
Empagliflozin	10.69 (9.01–12.62)	7.25 (6.26–8.46)	7.03 (6.10–8.06)	-20.89 ± 0.84	0.87 ± 1.20	0.469
Placebo	10.90 (9.20–12.83)	7.37 (6.25–8.74)	7.02 (5.71–8.53)	-20.02 ± 0.86		
NLR						
All	0.74 (0.67–0.80)	0.61 (0.56–0.68)	0.59 (0.53–0.66)	-10.22 ± 0.50		< 0.001
Empagliflozin	0.74 (0.67–0.80)	0.60 (0.56–0.67)	0.59 (0.53–0.66)	-9.69 ± 0.71	-1.11 ± 1.01	0.272
Placebo	0.74 (0.66–0.80)	0.62 (0.56–0.68)	0.60 (0.54–0.66)	-10.79 ± 0.72		
PLR						
All	125.58 (97.14–171.82)	117.00 (92.78–147.69)	115.38 (91.51–148.23)	-2.53 ± 0.92		0.006
Empagliflozin	127.38 (100.00–170.71)	113.96 (92.73–150.63)	115.10 (91.82–154.44)	-2.50 ± 1.29	-0.06 ± 1.84	0.974
Placebo	124.58 (93.04–173.33)	120.92 (94.00–146.91)	116.57 (91.13–145.38)	-2.56 ± 1.32		

*P-values are reported for the average percentage change in inflammatory markers from baseline to 26 weeks

*IQR, interquartile range; SD, standard deviation; Standard Error of Mean, SEM; hsCRP, high sensitive c-reactive protein; IL-6, interlukin-6; NLR, neutrophil-lymphocyte ratio, PLR; platelet-lymphocyte ratio*

### Primary endpoint

**Fig. 1:**
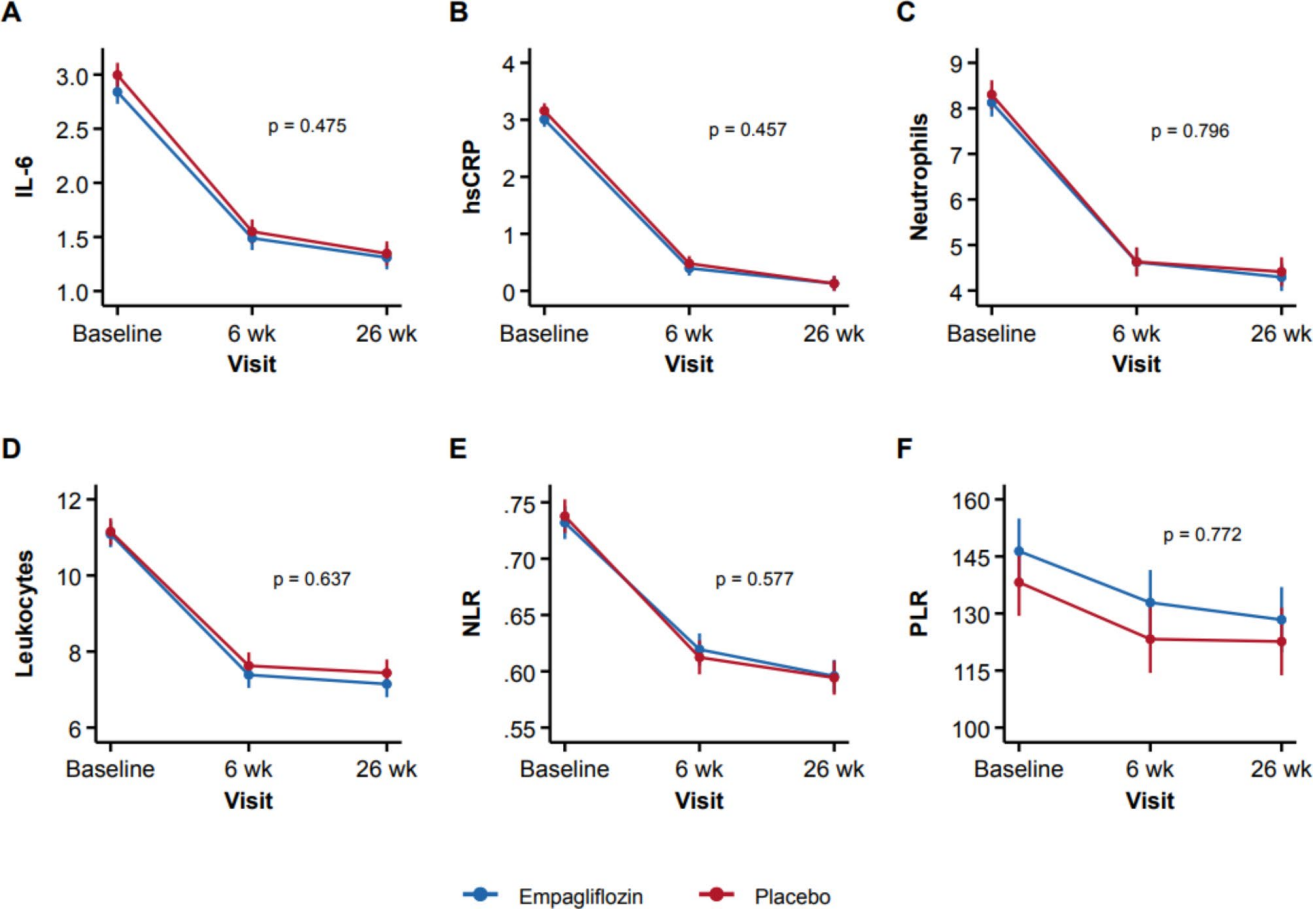
Mean ± SEM change in inflammatory markers over time by treatment *p = p-value for treatment-visit interaction, IL-6 and hsCRP values were log-transformed hsCRP, high sensitive c-reactive protein; IL-6, interlukin-6; NLR, neutrophil-lymphocyte ratio, PLR platelet to lymphocyte ratio

Inflammatory biomarkers decreased in both groups from baseline up to 26 weeks showing significant mean reduction of 35.1 ± 3.2% (p < 0.001) for IL-6, 57.4 ± 0.7% (p < 0.001) for hsCRP, 26.1 ± 0.7% (0.001) for neutrophils, 20.5 ± 0.6% (p < 0.001) for leukocytes, 10.2 ± 0.5% (p < 0.001) for NLR, and − 2.53 ± 0.92% for PLR (p = 0.006), but no significant difference between the Empagliflozin group and placebo was noted after 26 weeks. The reduction in inflammatory biomarkers occurred already at 6 weeks after AMI **(**Fig. 1**)**.

Median (IQR) 26-week IL-6 and hsCRP were numerically lower in the Empagliflozin group, but without a significant difference between both groups (p = 0.65 and p = 0.52, respectively). Likewise, neutrophils, leukocytes, NLR, and PLR were not significantly different between the groups at week 26 **(**Table 3**)**.

### Correlation analysis

Univariable linear mixed effect analysis of inflammatory biomarkers showed significant correlations of IL-6, hsCRP, and NLR with NTproBNP levels (p < 0.001) and high-sensitive troponin T (p < 0.001), LVEF was only observed to be significantly correlated with hsCRP (p < 0.001) and IL-6 (p < 0.001). Furthermore, IL-6 showed significant associations with diastolic function (p < 0.001) and hsCRP with body mass index (p < 0.001) **(**Table 4**)**.

**Table 4:** Univariable linear mixed effects model of inflammatory markers with clinical factors, renal, and cardiac markers

Markers	Log-IL-6		Log-hsCRP		NLR		PLR	
	**Coef** ± **SEM**	**p-value**	**Coef** ± **SEM**	**p-value**	**Coef** ± **SEM**	**p-value**	**Coef** ± **SEM**	**p-value**
Age	0.0004 ± 0.003	0.894	-0.002 ± 0.003	0.580	0.001 ± 0.001	0.011	1.23 ± 0.29	< 0.001
Sex (female/male)	0.062 ± 0.069	0.367	0.204 ± 0.082	0.014	0.014 ± 0.010	0.175	10.33 ± 0.703	0.141
BMI	0.020 ± 0.006	0.001	0.040 ± 0.007	< 0.001	0.001 ± 0.001	0.195	-1.11 ± 0.63	0.081
Diabetes (yes/no)	0.103 ± 0.077	0.182	0.042 ± 0.094	0.655	-0.008 ± 0.012	0.485	-20.82 ± 7.93	0.009
Smoking (yes/no)	0.076 ± 0.059	0.196	0.141 ± 0.071	0.046	-0.012 ± 0.009	0.150	-28.86 ± 5.82	< 0.001
Hyperlipidemia (yes/no)	0.004 ± 0.060	0.949	0.063 ± 0.073	0.387	-0.017 ± 0.009	0.055	-13.54 ± 6.09	0.026
Hypertension (yes/no)	0.130 ± 0.054	0.016	0.114 ± 0.065	0.081	-0.003 ± 0.008	0.710	0.05 ± 5.54	0.992
eGFR	-0.001 ± 0.001	0.471	0.001 ± 0.001	0.645	<-0.001 ± < 0.001	0.229	-0.12 ± 0.11	0.298
LVEF	-0.014 ± 0.003	< 0.001	-0.016 ± 0.004	< 0.001	< 0.001 ± < 0.001	0.512	0.27 ± 0.23	0.235
E/é	0.040 ± 0.010	< 0.001	0.027 ± 0.012	0.022	< 0.001 ± < 0.001	0.701	0.35 ± 0.72	0.622
NT-proBNP	0.224 ± 0.026	< 0.001	0.196 ± 0.031	< 0.001	0.013 ± 0.004	< 0.001	5.87 ± 2.03	0.004
Creatine kinase	0.125 ± 0.041	0.002	0.087 ± 0.048	0.071	-0.0004 ± 0.005	0.933	-2.32 ± 2.50	0.353
Troponin T	0.187 ± 0.040	< 0.001	0.226 ± 0.047	< 0.001	0.024 ± 0.005	< 0.001	2.91 ± 2.75	0.289

*BMI, body mass index; eGFR, estimated glomerular filtration rate; LVEF, left ventricular ejection fraction; NT-proBNP, N-terminal prohormone of brain natriuretic peptide;* hsCRP, high sensitive c-reactive protein; IL-6, interlukin-6; NLR, neutrophil-lymphocyte ratio; *SEM, Standard Error of Mean; PLR, platelet-lymphocyte ratio*

Those significant associations observed in simple LMEM were included in the multiple LMEM along with treatment, visit, treatment-visit interaction, age, sex, and diabetes.

In the multivariable linear mixed analysis, we found significant associations of hsCRP and IL-6 with Troponin T (p < 0.001) suggesting a direct association of increased inflammatory response after AMI and infarct size. Trajectories of inflammatory biomarkers after AMI did not differ between the Empagliflozin and placebo group. Moreover, hsCRP and IL-6 were significantly associated with NTproBNP (p < 0.001), E/E’ (p < 0.001) as well as LVEF (p < 0.001) over the observation period. BMI was significantly associated with hsCRP (p < 0.001). No treatment interaction was observed for any of the investigated inflammatory biomarkers in the multivariate model **(**Table 5**)**.

**Table 5:** Multivariable linear mixed effects model of inflammatory markers with clinical factors and cardiac markers

Markers	Coefficient	SEM	p-value	p-interaction
***IL-6***				
BMI	0.017	0.006	0.005	0.204
LVEF	− 0.01406	0.003	< 0.001	0.590
E/é	0.038	0.010	< 0.001	0.060
NT-proBNP	0.253	0.027	< 0.001	0.557
Creatine Kinase	0.140	0.077	0.001	0.996
Troponin T	0.202	0.040	< 0.001	0.145
***hsCRP***				
Sex (female/male)	0.265	0.077	0.001	0.991
BMI	0.03899	0.007	< 0.001	0.491
Smoking	0.151	0.069	0.030	0.647
LVEF	-0.016	0.003	< 0.001	0.112
E/é	0.023	0.012	0.042	0.386
NT-proBNP	0.220	0.032	0.008	0.768
Creatine Kinase	0.117	0.048	0.014	0.861
Troponin T	0.269	0.047	< 0.001	0.210
***NLR***				
Age	0.001	0.001	0.011	0.595
NT-proBNP	0.012	0.004	0.002	0.718
Troponin T	0.023	0.005	< 0.001	0.579
***PLR***				
Age	0.005	0.002	0.028	0.281
Diabetes	-0.121	0.056	0.030	0.426
Smoking	-0.163	0.044	< 0.001	0.297
Nt-proBNP	0.027	0.013	0.043	0.072

*p-interaction = p-value for treatment interaction with each variable

*BMI, body mass index; LVEF, left ventricular ejection fraction; NT-proBNP, N-terminal prohormone of brain natriuretic peptide;* hsCRP, high sensitive c-reactive protein; IL-6, interlukin-6; NLR, neutrophil-lymphocyte ratio; *SEM, Standard Error of Mean; PLR, platelet-lymphocyte ratio*

## Discussion

EMMY was the first clinical trial showing beneficial effects of Empagliflozin after AMI on cardiac biomarkers as well as structural and functional cardiac parameters compared to placebo when being administered within 72 h after PCI. Data of the SGLT2-I AMI Protect registry suggested anti-inflammatory effects as mediator of beneficial clinical outcome [25]. The post-hoc analysis showed elevated inflammatory biomarkers when initially presenting with AMI and showed a significant decrease up to 26 weeks, however, this effect was already evident at week 6. Nevertheless, the observed decline in inflammatory biomarkers did not differ significantly between Empagliflozin and the placebo group, when compared at week 26. 80% of the whole EMMY cohort had available blood samples of all three visits for this post-hoc analysis, however, the baseline characteristics were distributed equally in the EMMY trial as well as in the post-hoc group **(**Table 6**)**.

**Table 6:** Baseline characteristics in the entire EMMY cohort and analyzed sub-cohort

Characteristics	Entire EMMY cohort				EMMY cohort included in current analysis			
	All	Empagliflozin	Placebo	P-value	All	Empagliflozin	Placebo	P-value
All, n (%)	476	237 (49.79)	239 (50.21)	--	374	191 (51.07)	183 (48.93)	--
Sex, n (%)								
Male	392 (82.35)	195 (82.28)	197 (82.43)	0.966	305 (81.55)	160 (83.77)	145 (79.23)	0.258
Female	84 (17.65)	42 (17.57)	42 (17.72)		69 (18.45)	31 (16.23)	38 (20.77)	
Age (years), mean ± SD	57.66 ± 9.52	57.53 9.03	57.78 ± 10.01	0.774	57.56 ± 9.03	57.27 ± 8.67	57.87 ± 9.41	0.520
BMI (kg/m^2^), mean ± SD	27.98 ± 4.48	28.23 ± 4.22	27.72 ± 4.72	0.213	28.15 ± 4.26	28.24 ± 4.29	28.05 ± 4.24	0.676
Diabetes, n (%)	63 (13.24)	30 (12.66)	33 (13.81)	0.711	51 (13.64)	24 (12.57)	27 (14.75)	0.538
Systolic BP (mmHg), mean ± SD	127.00 ± 13.79	126.65 ± 14.74	127.34 ± 12.79	0.582	126.46 ± 13.37	126.30 ± 14.44	126.62 ± 12.19	0.815
Diastolic BP (mmHg), mean ± SD	80.18 ± 8.77	80.11 ± 8.83	80.25 ± 8.74	0.865	79.77 ± 8.25	80.11 ± 8.20	79.40 ± 8.30	0.406
Smoking (active or former), *n* (%)	341 (71.94)	171 (72.15)	170 (71.73)	0.919	267 (71.39)	138 (72.25)	129 (70.49)	0.707
Dyslipidemia, n (%)	135 (28.36)	71 (29.96)	64 (26.78)	0.442	102 (27.27)	61 (31.94)	41 (22.40)	0.039
Hypertension, n (%)	199 (41.81)	92 (38.82)	107 (44.77)	0.188	156 (41.71)	73 (38.22)	83 (45.36)	0.162
CAD, n (%)	53 (11.53)	28 (11.81)	25 (10.46)	0.639	30 (8.02)	19 (9.95)	11 (6.01)	0.161
Stroke, n (%)	6 (1.26)	5 (2.11)	1 (0.42)	0.121	5 (1.34)	4 (2.09)	1 (0.55)	0.372
ACS history, n (%)	23 (4.83)	14 (5.91)	9 (3.77)	0.276	16 (4.28)	11 (5.76)	5 (2.73)	0.148
PAD, n (%)	8 (1.68)	5 (2.11)	3 (1.26)	0.468	5 (1.34)	4 (2.09)	1 (0.55)	0.372
**Laboratory parameters**								
eGFR (mL/min/173m^2^), median (IQR)	92 (78–102)	92 (78–101)	91 (78–102)	0.883	92 (78–101)	93 (78–101)	90 (78–100)	0.679
Creatine kinase (U/L), median (IQR)	1673 (1202–2456)	1668 (1136–2532)	1701 (1254–2404)	0.71	1648 (1201–2452)	1596 (1126–2478)	1669 (1257–2417)	0.434
Troponin T (ng/L), median (IQR)	3039 (2037–4856)	3059 (2082–4775)	3029 (1980–4856)	0.56	3003 (2047–4647)	2947 (2062–4628)	3020 (1996–4871)	0.867
Total cholesterol (mg/dL), mean ± SD	191.93 ± 45.46	192.49 ± 45.16	191.40 ± 45.83	0.796	194.01 ± 45.27	193.56 ± 44.22	194.48 ± 46.45	0.847
LDL-cholesterol, (mg/dL), mean ± SD	121.69 ± 41.25	122.06 ± 40.00	121.33 ± 42.53	0.851	123.86 ± 40.17	123.88 ± 38.42	123.85 ± 42.04	0.995
HDL-cholesterol (mg/dL), median (IQR)	44 (36–54)	44 (36–52)	43 (36–54)	0.767	43 (36–52)	43 (36–52)	43 (36–52)	0.904
LVEF (%), median (IQR)	48 (43–53)	48 (43–53)	49 (43–54)	0.100	48 (43–54)	49 ± 7.53	48.9 ± 8.37	0.145
E/e‘, median (IQR)	8.94 (7.50–10.86)	8.94 (7.44–10.94)	8.94 (7.54–10.81)	0.609	9.06 (7.54–10.67)	9.13 (7.45–10.82)	9.04 (7.69–10.65)	0.563
NT-proBNP (pg/mL), median (IQR)	1294 (757–2246)	1273 (773–2249)	1373 (754–2217)	0.905	1365 (773–2192)	1271 (753–2127)	1436 (800–2217)	0.407
**Treatment**								
ACE-I/ARB, n (%)	459 (97.66)	228 (97.44)	231 (97.88)	0.749	361 (97.57)	186 (97.89)	175 (97.22)	0.675
Beta-blocker, n (%)	457 (96.41)	223 (94.89)	234 (97.91)	0.078	360 (96.26)	181 (94.76)	179 (97.81)	0.120
MRA, n (%)	180 (37.39)	86 (36.60)	94 (39.33)	0.540	143 (38.24)	70 (36.65)	73 (39.89)	0.519
Statin, n (%)	462 (97.47)	229 (97.45)	233 (97.49)	0.976	368 (98.40)	187 (97.91)	181 (98.91)	0.441
Ezetimibe, n (%)	59 (12.45)	29 (12.34)	30 (12.55)	0.944	43 (11.50)	23 (12.04)	20 (10.93)	0.736
Platelet inhibitory drugs, n (%)	476 (100.00)	237 (100.00)	239 (100.00)	1.000	374 (100.00)	191 (100.00)	183 (100.00)	1.000
Anticoagulation drugs, n (%)	37 (7.79)	16 (6.78)	21 (8.79)	0.414	26 (6.95)	11 (5.76)	15 (8.20)	0.354
Metformin, n (%)	41 (8.63)	21 (8.90)	20 (8.37)	0.837	37 (9.89)	17 (8.90)	20 (10.93)	0.511
GLP1-RA, n (%)	4 (0.84)	2 (0.85)	2 (0.84)	0.990	3 (0.80)	1 (0.52)	2 (1.09)	0.537

*BMI, body mass index; BP, blood pressure; CAD, coronary artery disease; AMI, acute myocardial infarction; PAD, peripheral artery disease; eGFR, estimated glomerular filtration rate; LVEF, left ventricular ejection fraction; NT-proBNP, N-terminal prohormone of brain natriuretic peptide; SD, standard deviation; IQR, interquartile range, LDL, low-density lipoprotein; HDL, high-density lipoprotein; ACE-I, angiotensin-converting enzyme inhibitor; ARB, angiotensin receptor blocker; MRA, mineralocorticoid receptor antagonist; GLP1-RA, glucagon-like peptide 1 receptor agonist*

Systemic vascular inflammation plays a pivotal role in the progression and destabilization of atherosclerotic cardiovascular disease by inducing atheroprogression in a stable setting, initiating atheroma destabilisation provoking AMI as well as responding to myocardial necrosis with cardiac remodeling [9, 10, 35, 36]. Biomarkers like hsCRP and interleukins play an important role in the inflammatory process of atherosclerosis [1, 5, 37] and show a significant increase after initial presentation with AMI [6, 7, 9, 10].

SGLT2-inhibitors were found to exert beneficial effects on inflammatory biomarkers such as hsCRP and IL-6 compared to other glucose-lowering agents, and thus attenuating low-grade inflammation, a well-known key driver of vascular complications [38].

The only available data regarding the effects of SGLT2-inhibitors on inflammatory biomarkers in acute myocardial infarction derive from the SGLT2-I AMI Protect international registry, which investigated the impact of chronic SGLT2-I treatment on inflammatory biomarkers in patients with diabetes presenting with AMI. This registry reports significantly lower baseline levels of leucocytes, neutrophils, and hsCRP in patients treated with SGLT2-I [25]. The multivariable analysis highlights the use of SGLT2-inhibitors as a significant predictor of reduced inflammatory response after AMI. Conversely, peak troponin values and NSTEMI occurrence turned out to be independent predictors of higher inflammatory status [25]. Of note, the SGLT2-I AMI PROTECT trial reported normal baseline HbA1c levels in the SGLT2-I and non SGLT2-I group suggesting that SGLT2-I treatment lowers diabetes-induced inflammation [39] independent of glucose lowering effect. In our analysis we found highly elevated initial inflammatory biomarkers at baseline in both groups without associations to diabetes in the univariable analysis. This highlights a positive effect of SGLT2-I on inflammatory biomarkers in the SGLT2-I AMI PROTECT trial being a chronic treatment effect with SGLT2-I. However, no data obtained from randomised controlled clinical trials have been published elucidating the effects of SGLT2-I on inflammatory biomarkers in AMI when added to post-MI guideline-recommended treatment.

In the acute phase of myocardial infarction, hsCRP is significantly higher compared to controls and positively correlated with the severity of coronary lesions and is an independent predictor for systolic and diastolic cardiac function [40–42]. Further, patients with an initially increased hsCRP were at higher risk of major adverse cardiovascular events, cardiovascular death, and all-cause death [6, 7, 43].

Similar results were found for interleukin-6 showing inverse correlations with systolic and diastolic function [40], and IL-6 was observed to be independently correlated to larger infarct size, reperfusion injury, and higher likelihood for left ventricular remodeling [9, 10, 43, 44]. In this subgroup-analysis of the EMMY trial, we found highly significant correlations of hsCRP and IL-6 with troponin T in multivariable linear mixed analysis, suggesting an increased inflammation in larger AMI. However, inflammatory biomarker trajectories did not differ between empagliflozin and placebo. Moreover, hsCRP and IL-6 were significantly associated with NTproBNP, E/E’ as well as LVEF indicating a direct relation with infarct size and disease severity. Empagliflozin has been shown to impact AMPK-mediated pathways and TNFa induction in cell models with and without lipopolysaccharide-induced inflammation [45]; however, no influence on hsCRP and IL-6 has been observed so far.

The neutrophil-to-lymphocyte ratio (NLR) also demonstrated greater levels after initial presentation with AMI and was significantly positively related to troponin T levels, in line with data showing NLR to be associated with myocardial dysfunction [17], cardiac remodeling [46] as well as being a predictor for myocardial damage/necrosis [17, 47], long term prognosis [18, 48], mechanical complications [49], thrombus burden [50, 51], and procedural complications [52]. Patients with diabetes, receiving SGLT2-I, were identified to have lower initial NLR in AMI as well as smaller infarct size [25]; however, the EMMY sub-analysis did not exhibit such differences in NLR between Empagliflozin and placebo group post-MI.

Leukocyte and neutrophil count were observed to be elevated in AMI showing positive correlations with peak Troponin T, infarct size as well as LVEF and therefore are independent predictors for cardiovascular outcome [14–16], but only neutrophil count was identified as an independent predictor for high thrombus burden as well as total coronary occlusion [53] and no-reflow following primary PCI in STEMIs [54]. The trajectories of leukocyte and neutrophil count in the EMMY sub-analysis reflects inflammatory peaks in AMI as an excerpt of a systemic inflammatory process followed by cardiac remodeling.

The PLR as a suggested predictor for cardiovascular outcome [19, 20, 22] has shown to be significantly correlated with age and is associated with poor in-hospital outcome of elderly patients with AMI suggesting that inflammation and prothrombotic state may contribute to these patients [19, 55]. Further, we found significant associations of the smoking status with PLR in multivariable analysis suggesting this to be a predictor for morbidity [56] and higher thrombus burden [51] in AMI patients.

In the EMMY subgroup-analysis, the greater excursion of baseline inflammatory parameters was associated with larger infarct size as well as decreased cardiac function with a pronounced decline up to 26 weeks. The results demonstrate that inflammation is a physiological reaction leading to cardiac fibrosis to facilitate the healing process of damaged myocardium [57]. Thus, inflammation plays a crucial role in ventricular cardiac remodeling [58] and numerous reactants as well as immune cells are involved in this complex process [13, 57–60], but no difference in trajectories of inflammatory biomarkers between the SGLT2-inhibitor Empagliflozin and placebo was observed in the EMMY subgroup-analysis.

### Strengths and study limitations

In this post-hoc analysis, frozen biomarker samples of 374 patients were available for complete analysis (80% of the whole cohort). Moreover, the EMMY trial was not powered for hard clinical endpoints due to the low number of patients and short follow-up period. Larger sample sizes and a longer follow-up period would be necessary to identify relevant associations to hard clinical endpoints. However, two large, adequately powered randomized controlled clinical trials (EMPACT-MI [NCT04509674] and DAPA-MI [NCT04564742]), are eagerly awaited to fill this gap in knowledge. Nonetheless, these trials might not be able to provide detailed data on inflammatory markers and their trajectories.

Furthermore, in EMMY sex groups were not balanced in both groups and the percentage of diabetic patients was smaller than expected. But both factors did not demonstrate to have a potential impact on trajectories of inflammatory biomarkers in univariable linear mixed analysis.

Pro-resolving mediators might play a role in the inflammatory burden as well as clinical outcome post-AMI, however, analysis of these would be beyond the scope of this analysis.

## Conclusion

The results of the recently published EMMY trial presented first evidence for the administration of SGLT2-I after AMI in addition to guideline-recommended post-MI therapy showing significant reduction in NTproBNP levels at weeks 26 compared to placebo independent of diabetic status. In this post-hoc analysis, a great extend in inflammatory biomarkers was identified at initial presentation with AMI with a significant decline up to week 26 that was already evident at week 6. However, no difference between SGLT2-I and placebo was observed with regards to inflammatory biomarkers suggesting that inflammatory response post-MI is not significantly altered by Empagliflozin.

## Data Availability

Not applicable.
